# A Systematic Review and Meta-Analysis on Neural Adaptations Following Blood Flow Restriction Training: What We Know and What We Don't Know

**DOI:** 10.3389/fphys.2020.00887

**Published:** 2020-08-04

**Authors:** Christoph Centner, Benedikt Lauber

**Affiliations:** ^1^Department of Sport and Sport Science, University of Freiburg, Freiburg, Germany; ^2^Department of Neurosciences and Movement Sciences, University of Fribourg, Fribourg, Switzerland

**Keywords:** blood flow restriction, neural adaptations, muscle excitation, kaatsu, resistance training

## Abstract

**Objective:** To summarize the existing evidence on the long-term effects of low-load (LL) blood flow restricted (BFR) exercise on neural markers including both central and peripheral adaptations.

**Methods:** A systematic review and meta-analysis was conducted according to the PRISMA guidelines. The literature search was performed independently by two reviewers in the following electronic databases: PubMed, Web of Science, Scopus and CENTRAL. The systematic review included long-term trials investigating the effects of LL-BFR training in healthy subjects and compared theses effects to either LL or high-load (HL) training without blood flow restriction.

**Results:** From a total of *N* = 4499 studies, *N* = 10 studies were included in the qualitative synthesis and *N* = 4 studies in a meta-analysis. The findings indicated that LL-BFR resulted in enhanced levels of muscle excitation compared to LL training with pooled effect sizes of 0.87 (95% CI: 0.38–1.36). Compared to HL training, muscle excitation following LL-BFR was reported as either similar or slightly lower. Differences between central activation between LL-BFR and LL or HL are less clear.

**Conclusion:** The summarized effects in this systematic review and meta-analysis highlight that BFR training facilitates neural adaptations following LL training, although differences to conventional HL training are less evident. Future research is urgently needed to identify neural alterations following long-term blood flow restricted exercise.

## Introduction

The need of optimizing walking ability and specific motor skills is an important challenge in neurologic and orthopedic rehabilitation. Following injury or surgical interventions (e.g., ACL reconstruction) patients are often prescribed with an individualized period of limb or even whole-body immobilization (Hiemstra et al., 2006). During this period of mechanical unloading, individuals frequently show a rapidly decreasing neuromuscular control (Campbell et al., 2019) as well as pronounced skeletal muscle atrophy (Psatha et al., 2012).

In order to increase muscle mass, resistance training with high training loads (70–85% of the individual one repetition maximum, 1RM) is generally recommended (ACSM, 2009) although a high exposure to mechanical stress on joints is often contraindicated in many fields of rehabilitation. In recent years, blood flow restriction (BFR) training has been shown to induce beneficial adaptations in muscle mass and strength even when the applied loads are only around 15–20% 1RM (Jessee et al., 2018). BFR training involves the application of a tourniquet/cuff at the most proximal portion of the training limb (Patterson et al., 2019) leading to an increased metabolic accumulation (Takada et al., 2012) and growth hormone release (Takarada et al., 2000). Comparing the adaptive responses of low-load BFR (LL-BFR) training to conventional high-load (HL) training, two recent systematic reviews and meta-analyses have indicated that adaptations on the structural level (e.g., muscle hypertrophy) are similar (Lixandrao et al., 2018; Centner et al., 2019b). However, it seems that strength gains are more pronounced when training with heavy loads (Lixandrao et al., 2018; Centner et al., 2019b). According to a theory proposed by Sale (1988), increases in strength are mediated by either neural adaptations or muscular hypertrophy or a combination of both. Since the latter seems comparable between LL-BFR and HL training modalities (Lixandrao et al., 2018; Centner et al., 2019b), it might be speculated that both regimens differ in their neuromuscular response. Although a small number of previous investigations have already examined the magnitude of neuromuscular responses following LL-BFR training, these findings are conflicting with some studies reporting no differences between HL and LL-BFR (Sousa et al., 2017; Cook et al., 2018) and another study showing higher neural activation when training with HL only (Kubo et al., 2006). Similar inconsistent findings have been observed when comparing LL training with LL-BFR training (Thiebaud et al., 2014; Lauver et al., 2017; Oranchuk et al., 2019). This second comparison (LL vs. LL-BFR) is not less important, considering the common implementation of low-load regimens in clinical settings especially during the early phases of rehabilitation (Bousquet et al., 2018).

While the investigation of short-term responses in neural parameters is crucial for studying basic mechanisms, the long-term and chronic adaptations are of exceptional relevance for both clinical rehabilitation and sports. Especially for healthy athletes who aim for implementing BFR training in their training routine, a more detailed understanding of neural responses is essential for optimizing training adaptations. A lack of neural adaptations would question the functionality of exercises with blood flow restriction. Therefore, the main objective of this systematic review was to summarize the existing evidence on the long-term (≥ 4 weeks) effects of LL-BFR exercise on neural markers (peripheral vs. central) compared to HL and LL training without BFR.

## Methods

### Protocol and Registration

The present systematic review was performed in accordance with the PRISMA guidelines (Moher et al., 2009) on the basis of a prospectively defined review protocol submitted to PROSPERO.

### Search Strategy and Study Identification

A systematic literature search was completed in the following electronic databases from inception to 1st February 2020: PubMed, Web of Science, Scopus, and CENTRAL. Keywords included terms associated with blood flow restriction training which were connected with terms for neuromuscular outcomes. The search was performed with no restrictions and the final search string took the following form:

“blood flow restriction” OR “occlusion training” OR “vascular occlusion” OR KAATSU OR “ischemi^*^ training” AND “neural” OR “neuronal” OR “musc^*^ activ^*^” OR “myoelectric^*^” OR excitability OR neuromuscular OR reflex OR “motor unit” OR TMS

**Figure 1:**
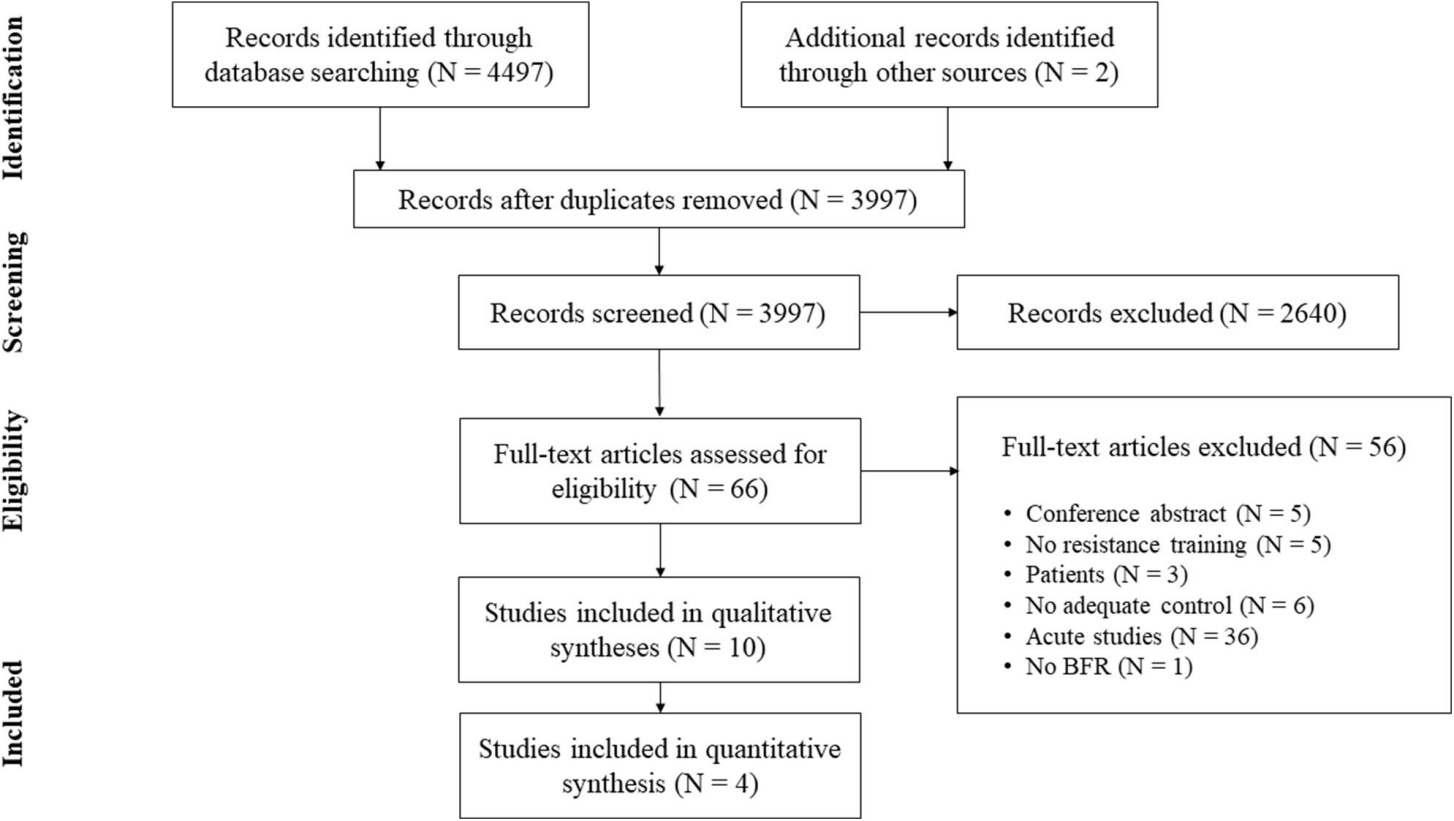
Flow chart.

In addition to database searching, the reference lists of the obtained articles were screened for further eligible papers (Horsley et al., 2011). All studies were stored in a citation manager and duplicates were removed before further processing (for search process see Figure 1).

### Eligibility Criteria

All articles were independently screened by two reviewers (CC & BL) according to the following PICOS-conform inclusion criteria: (i) healthy adult humans (18+ years); (ii) LL-BFR training (≤ 50% 1RM/MVC) was compared to a control group receiving either LL training without BFR (≤ 50% 1RM/MVC) or HL training (> 50% 1RM/MVC); (iii) neuromuscular parameters were assessed before and after a long-term intervention (≥ 4 weeks of training).

Studies were excluded from this systematic review if (i) experiments were performed on animals or patients; (ii) the manuscript was written in a language other than English; (iii) published abstracts or conference proceedings; (iv) study quality was rated with a score <6 using the Physical Evidence Database (PEDro) scale (Verhagen et al., 1998). The PEDro scale is composed of 11 items and all studies were independently rated by two reviewers (CC & BL) (see Table 1).

**Table 1:** Study quality assessment with the Physical Evidence Database (PEDro).

**References**	**Study quality criterion**										
	**2**	**3**	**4**	**5**	**6**	**7**	**8**	**9**	**10**	**11**	**Total**
Biazon et al. (2019)	1	0	1	0	0	0	1	1	1	1	6
Colomer-Poveda et al. (2017)	1	0	1	0	0	0	1	1	1	1	6
Cook et al. (2018)	1	0	1	0	0	0	1	1	1	1	6
de Castro et al. (2019)	1	0	1	0	0	0	1	1	1	1	6
Hill et al. (2020)	1	0	1	0	0	0	1	1	1	1	6
Kubo et al. (2006)	1	0	1	0	0	0	1	1	1	1	6
Manimmanakorn et al. (2013)	1	0	1	0	0	1	1	1	1	1	7
Moore et al. (2004)	1	0	1	0	0	0	1	1	1	1	6
Ramis et al. (2020)	1	0	1	0	0	1	1	0	1	1	6
Sousa et al. (2017)	1	0	1	0	0	0	1	1	1	1	6

### Data Extraction and Collection

After an initial screening, all retained articles were considered relevant and the following information was extracted after accessing the full-texts: (i) population characteristics, (ii) exercise/training protocol specifics, (iii) methodological approach, (iv) main findings.

In case of multiple time point assessments during interventions, only the first and very last time point was considered. Due to a high variability in EMG data, data extraction was conducted in a prioritized fashion (Gronfeldt et al., 2020). Therefore, EMG data derived from the lower extremity muscles were prioritized over upper extremity muscles. Data from lower extremity muscles were extracted in the following order: vastus lateralis (VL) > vastus medialis (VM) > rectus femoris (RF) > soleus (SOL). EMG data extraction in the upper limbs were prioritized in the following order: biceps brachii > triceps brachii > brachioradialis. In case of unavailable raw data, the corresponding author of the manuscript was contacted. If the respective authors could not be reached, data were extrapolated from figures using ImageJ software [NIH, Maryland, USA].

All data were independently extracted and screened by two researchers (CC & BL). In case of disagreement (*n* = 1), consensus was found in one of the regular discussion meetings.

### Risk of Bias Assessment

Risk of bias within each included study was assessed by the Cochrane Collaboration's Tool (Higgins and Green, 2011). Therefore, selection bias, performance bias, detection bias, attrition bias and reporting bias were evaluated (Supplementary Material 1). In this context, the risk of bias was ranked as either “adequate,” “unclear,” or “inadequate” (Higgins and Green, 2011). For evaluating the risk of bias across studies, publication bias was considered by visually inspection of funnel plots (LL-BFR vs. LL: Supplementary Material 2; LL-BFR vs. HL: Supplementary Material 3).

### Synthesis of Results and Statistical Approach

If enough data for a quantitative approach were available, the standardized mean difference (SMD) was calculated. If standard deviation from a study was not reported, this was estimated from standard error or confidence intervals according to the Cochrane Handbook of Systematic Reviews (Higgins and Green, 2011). Due to high differences in standard deviation between time points, SD_change_ was defined as: root square [(SD_pre_^2^/N_pre_) + (SD_post_^2^/N_post_)] (Borenstein et al., 2009). All meta-analyses were performed using a random-effects model with inverse variance weighting. In addition, the impact of the inconsistency and heterogeneity across studies on the meta-analysis was evaluated with the *I*-squared method. *I*-squared was calculated as: ((chi-squared statistic—degrees of freedom)/chi-squared statistic) × 100% (Higgins and Green, 2011). In accordance to the Cochrane Handbook of Systematic Reviews of Interventions (Higgins and Green, 2011), *I*-squared was interpreted as follows: 0–40% representing a low heterogeneity, 30–60% representing a moderate heterogeneity, 50–90% a substantial, and 75–100% a considerable heterogeneity.

Due to the fact that a considerable amount of studies incorporated a within-subject study design (e.g., left vs. right leg) and that this facilitates a smaller variance within a single study compared to a parallel group design, only one meta-analysis was performed for studies including parallel-group designs. Additionally, within-subject designs are frequently biased by the cross-education effect, which might further influence their results in training studies (Lee and Carroll, 2007).

## Results

From initially 4,499 studies, we assessed the full-texts of 66 studies (for full search process see Figure 1). After checking for eligibility of these articles based on our inclusion and exclusion criteria, 56 studies were excluded. At the final stage, at total of *n* = 10 studies with *n* = 232 participants were included in the systematic review (Table 2; Moore et al., 2004; Kubo et al., 2006; Manimmanakorn et al., 2013; Colomer-Poveda et al., 2017; Sousa et al., 2017; Cook et al., 2018; Biazon et al., 2019; de Castro et al., 2019; Hill et al., 2020; Ramis et al., 2020).

**Table 2:** Overview of studies included in the systematic review.

**References**	**Subjects**	**Protocol**	***N***	**Pressure modality**	**Exercise mode**	**Duration/** **frequency**	**Neurophysiological parameters**	**Conclusion**
Biazon et al. (2019)	Untrained men	LL-BFR (20% 1RM) HL (80% 1RM) HL + BFR (80% 1RM)	30^#^	Intermittent BFR (60% AOP) Cuff width: 17.5 cm	Unilateral leg extension	10 weeks; 2 times/wk	- MVC normalized RMS EMG	- Similar EMG amplitude following LL-BFR compared to HL training (over time) - Generally higher EMG amplitudes in HL compared to LL-BFR (condition effect)
Colomer-Poveda et al. (2017)	Recreationally active men	LL-BFR (25% MVC) LL (25% MVC) CON (no training)	7 7 8	N/A (150–210 mm Hg) Cuff width: 13cm	Unilateral isometric plantar flexion	4 weeks; 3 times/wk	- V Waves - M Waves - H Reflex - M_max_ - V_wave_/M_wave_ ratio - H_max_/M_max_ ratio - iEMG/M_max_ ratio - EMG absolut (data from author)	No significant changes in any of the groups
Cook et al. (2018)	Untrained men and women	LL-BFR (20% 1RM) HL (70% 1RM) CON (no training)	6 6 6	Continuous BFR (180–200 mm Hg) Cuff Width: 5.4cm	Knee extension Leg press	6 weeks; 3 times/wk	- Central activation ratio (%) - Twitch Torque - Doublet torque - PAP - HRT	No significant changes in any of the groups
de Castro et al. (2019)	Resistance trained males	LL-BFR (20% 1RM) LL (20% 1RM)	11 9	Intermittent BFR (100% AOP) (Cuff width: 15cm)	Unilateral knee extension	6 weeks; 2 times/wk	- Median Frequency - RMS EMG	No change in RMS EMG and median frequency between baseline and post 6 weeks in LL and LL-BFR
Hill et al. (2020)	Recreationally active women	LL-BFR (30% peak torque) LL (30% peak torque) CON (no training)	10 10 10	Continuous BFR (40% AOP) Cuff width: 3cm	-Forearm–flexion -Forearm extension	4 weeks; 3 times/wk	- MVC normalized RMS EMG - Mean power frequency	- Similar increases in mean power frequency in LL-BFR and LL - No significant differences in EMG between LL-BFR and LL
Kubo et al. (2006)	Healthy young men	LL-BFR (20% 1RM) HL (80% 1RM)	9^#^	Continuous BFR (~180–240 mm Hg) Cuff width: N/A	Unilateral knee extension	12 weeks; 3 times/wk	- IEMG - Voluntary/Central activation	Significant increase in HL group, no significant increase in LL-BFR group
Manimmanakorn et al. (2013)	Female athletes	LL-BFR (20% 1RM) LL (20% 1RM) HL (80% 1RM) + hypoxic air	10 10 10	Continuous (BFR) (160–230 mm Hg) Cuff width: 5cm	Bilateral knee extension Bilateral knee flexion	5 weeks; 3 times/wk	- MVC normalized RMS EMG during maximal tasks	- Significant increase in EMG in LL-BFR and LL - Significantly higher EMG amplitudes in LL-BFR compared to LL
Moore et al. (2004)	Untrained young men	LL-BFR (50% 1RM) LL (50% 1RM)	8^#^	Continuous BFR (100 mm Hg) Cuff width: 7cm	Unilateral elbow flexion	8 weeks; 3 times/wk	- Central activation - PAP - HRT - Twitch:MVC ratio - IEMG - Twitch Torque	- Resting twitch decreased in LL-BFR but not LL - twitch:MVC ratio decreased in LL-BFR but not LL - PAP was increased in LL-BFR compared to LL following training - No differences were seen for HRT
Ramis et al. (2020)	Young and active men	LL-BFR (30% 1RM) HL (80% 1RM)	15 13	Continuous BFR (Arm: SBP – 20 mm Hg; Leg: SBP + 40 mm Hg) Cuff width: 14cm	- Unilateral elbow flexion - Unilateral knee extension	8 weeks; 3 times/wk	- RMS EMG during maximal tasks	Significant increase in EMG amplitude in both HL (biceps) and LL-BFR (vastus lateralis).
Sousa et al. (2017)	Untrained young individuals	LL-BFR (30% 1RM) HL (80% 1RM) LL-BFR + HL (30 + 80% 1RM) LL (30% 1RM)	10 11 8 8	Continuous BFR (80% AOP) Cuff width: 18cm	Unilateral knee extension	6 weeks; 2 times/wk	- MVC normalized RMS EMG - Median frequency	All groups showed an increased EMG amplitude and median frequency which was not significantly different between the groups

# *Within-subject design; 1RM, one-repetition maximum; AOP, Arterial Occlusion Pressure; CON, control group; EMG, electromyography; HL, high-load; HRT, Half-relaxation time; LL, Low-Load; LL-BFR, Low-load blood flow restriction; MVC, maximum voluntary contraction; N/A, not applicable; PAP, post-activation potentiation; RMS, root mean square; SBP, systolic blood pressure; wk, week*.

### Methodological Approaches

In the majority of included studies, neural changes were estimated by surface electromyography (sEMG) (Moore et al., 2004; Kubo et al., 2006; Manimmanakorn et al., 2013; Colomer-Poveda et al., 2017; Sousa et al., 2017; Biazon et al., 2019; de Castro et al., 2019; Hill et al., 2020; Ramis et al., 2020) which allows conclusion about the excitation state of the respective muscle. Additionally, median and mean power frequency were computed to investigate shiftings in the sEMG power spectrum (Sousa et al., 2017; de Castro et al., 2019; Hill et al., 2020). For further insights into corticospinal adaptations, three studies measured central activation (Moore et al., 2004; Kubo et al., 2006; Cook et al., 2018) by means of the twitch-interpolation technique. Two of these studies additionally assessed twitch torque, post-activation potentiation and half-relaxation time (Moore et al., 2004; Cook et al., 2018). Only one study reported changes in spinal excitability by assessing H-reflexes and V-waves (Colomer-Poveda et al., 2017).

### LL-BFR vs. LL

#### Muscle Excitation

Changes in muscle excitation and EMG spectral parameters were evaluated in five studies (Manimmanakorn et al., 2013; Colomer-Poveda et al., 2017; Sousa et al., 2017; de Castro et al., 2019; Hill et al., 2020). The majority of studies incorporated 12 training sessions (Colomer-Poveda et al., 2017; Sousa et al., 2017; de Castro et al., 2019; Hill et al., 2020) and one study 15 training sessions (Manimmanakorn et al., 2013). Investigated muscles within the studies included the individual muscles VM, VL (de Castro et al., 2019), SOL (Colomer-Poveda et al., 2017), and biceps brachii (Hill et al., 2020) as well as a calculated average of quadriceps muscles (Manimmanakorn et al., 2013; Sousa et al., 2017). While studies found no statistically significant differences in EMG amplitude between LL-BFR and LL (Colomer-Poveda et al., 2017; Sousa et al., 2017; de Castro et al., 2019; Hill et al., 2020) only the study from Manimmanakorn et al. (2013) with ~20% more training sessions demonstrated an augmented muscle activation with LL-BFR. The reason why four studies did not reveal significant findings might be explained by the limited power. These studies (Colomer-Poveda et al., 2017; Sousa et al., 2017; de Castro et al., 2019; Hill et al., 2020) included small sample sizes of *n* = 7–11 but found descriptive changes in EMG amplitude of 8–80% following LL-BFR and −11%–+25% following LL training. Regarding median frequency, two studies reported a significant time effect (Sousa et al., 2017; Hill et al., 2020), but no interaction effect.

**Figure 2:**
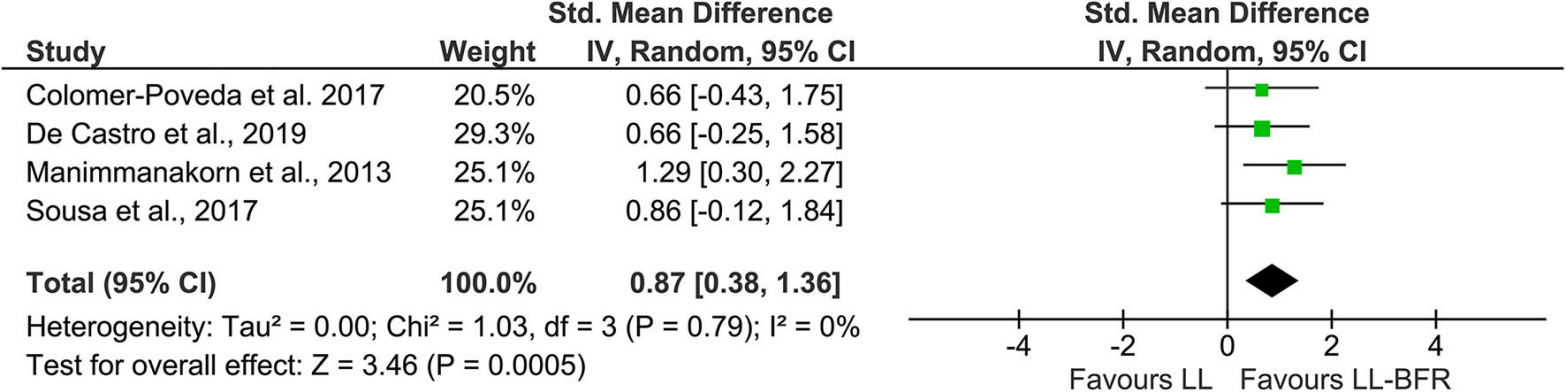
Forest plot depicting the pooled effect of low-load blood flow restriction training **(Right)** vs. low-load training **(Left)** on muscle excitation. CI, confidence interval; LL, low-load; IV, inverse variance; LL-BFR, low-load blood flow restriction; Random, random effects model.

In order to increase statistical power and create a more precise estimate of the effect on EMG amplitude, the results of the primary sources were pooled in a quantitative meta-analytical approach. In this context, *n* = 4 studies with parallel-group design were included in this meta-analysis (Figure 2). Due to the unavailability of raw data, it was not possible to include the study from Hill et al. (2020). The calculation revealed a significant (*Z* = 3.46, *p* < 0.01) pooled effect size of 0.87 (95% CI: 0.38–1.36) in favor of LL-BFR. Heterogeneity determined by *I*-squared was very low (*I*-squared = 0%, *p* = 0.79).

#### Central Activation

Overall, two studies investigated the effects of LL-BFR training on central and spinal variables and compared these to the same training without BFR (Moore et al., 2004; Colomer-Poveda et al., 2017). Both trials included young and male participants. Colomer-Poveda et al. (2017) conducted a 4-week training period with an unilateral training of the plantar flexors with alternating isometric contractions (25% MVC). Following the completion of 12 training sessions, the authors found no increases in H-reflex excitability or V-wave activity in any of the two groups (LL-BFR vs. LL) during rest and activity.

A study by Moore et al. (2004), implemented a training intervention being twice as long (8 weeks, 24 sessions) and assessed central activation by means of twitch-interpolation and found a decreased resting twitch and twitch:MVC ratio for LL-BFR, which was not evident in the LL group following 8 weeks of elbow flexion training. An increase in post-activation potentiation occurred in the LL-BFR compared to the LL group. However, half-relaxation time did not significantly change in any of the two groups.

### LL-BFR vs. HL

#### Muscle Excitation

As a measure for muscle excitation, EMG amplitude was determined in four studies (Kubo et al., 2006; Sousa et al., 2017; Biazon et al., 2019; Ramis et al., 2020). In three studies, EMG amplitude was reported to be increased following both LL-BFR and HL training (Sousa et al., 2017; Biazon et al., 2019; Ramis et al., 2020). The findings from Kubo et al. demonstrated an augmented EMG response only for HL (Kubo et al., 2006). Although no interaction effects were reported, Biazon et al. (2019) found a significantly higher overall EMG amplitude for HL compared to LL-BFR (condition effect).

**Figure 3:**
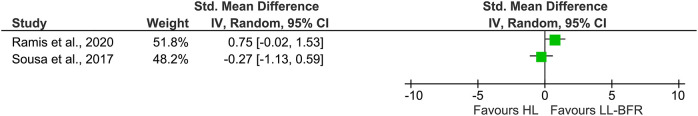
Forest plot illustrating the standardized mean differences of two studies describing the effects of HL **(Left)** and LL-BFR training **(Right)** on muscle excitation using a parallel group design. The pooled effect size was not included due to a high heterogeneity. CI, confidence interval; HL, high-load; IV, inverse variance; LL-BFR, low-load blood flow restriction; Random, random effects model.

While a quantitative evaluation of EMG amplitudes across studies was not possible due to the small amount of studies and the different study designs (parallel vs. within-subject), a descriptive presentation of the single effects is given in Figure 3. The studies by Biazon et al. (2019) as well as Kubo et al. (2006) were not included in Figure 3 because of their within-subject design. Additionally, the pooled effect size was omitted to avoid misleading interpretation of the results.

#### Central Activation

The effects of LL-BFR and HL training on corticospinal responses were compared in only two studies (Kubo et al., 2006; Cook et al., 2018). Although their results in central activation are numerically comparable (HL: ~ +3%; LL-BFR: ~ −2%), only Kubo et al. found a significant increase of central activation in HL, with no changes in LL-BFR (Kubo et al., 2006). The study from Cook et al. (2018) revealed that after 6 weeks of knee extension and leg press training, neural responses (central activation, twitch torque parameters, post-activation potentiation, and half-relaxation time) did not significantly change in any of the groups.

## Discussion

The aim of this systematic review was to identify and quantify neural adaptations after low-load blood flow restriction training and compare these effects to low-load and high-load resistance training without BFR. With a quantitative meta-analytic approach, it was shown that low-load resistance training with blood flow restriction as opposed to low-load training without BFR leads to an increased EMG activity (Figure 2) while the differences between HL and LL-BFR are less clear (Figure 3). With reference to changes in central activation, the qualitative review revealed that evidence is still far from conclusive with only a small number of studies and conflicting results which preclude from drawing definite conclusions.

### LL-BFR vs. LL

#### Muscle Excitation

The results regarding the differences in muscle excitation between LL and LL-BFR training demonstrated a significant increase in surface muscle EMG in favor of LL-BFR. The longitudinal changes which are reviewed in this work, resemble findings from cross-sectional studies looking at the acute effects of training with partial vascular occlusion (Kinugasa et al., 2006; Lauver et al., 2017, 2019; Husmann et al., 2018; Ilett et al., 2019; Kjeldsen et al., 2019). In a frequently cited study by Moritani et al. (1992) participants were instructed to complete a 4-min bout of repeated intermittent isometric hand grip contractions (20% MVC) with one group having the blood flow restricted during the second minute (cuff pressure 200 mmHg). The authors found that during the LL-BFR condition, RMS EMG amplitudes almost doubled and were significantly higher than in the LL condition. These differences were still apparent after the end of the 4-min period. Since then, similar observations were reported by several research groups (Kinugasa et al., 2006; Lauver et al., 2017, 2019; Husmann et al., 2018; Ilett et al., 2019; Kjeldsen et al., 2019). Even though the results of the latter studies indicated profound increases in muscle excitation with LL-BFR, there was also a small number of studies which could not find any between-group differences (Thiebaud et al., 2014; Oranchuk et al., 2019). An important factor that needs to be considered for the interpretation of these results is the applied exercise protocol as it has been demonstrated that exercising to volitional fatigue mitigates the differences seen between LL and LL-BFR when performed under non-fatiguing conditions (Wernbom et al., 2009; Cook et al., 2013).

In this context it needs to be highlighted, however, that all the studies presented here and in the present review used bipolar surface EMG to measure muscle excitation. This can be problematic as changes in EMG cannot unambiguously be attributed to changes in motor unit (MU) recruitment and firing because additional influences such as muscle fiber potential, motor unit synchronization and fatigue can influence the EMG amplitude (Wernbom and Aagaard, 2020). With the course of technological advancements, Fatela et al. (2019) non-invasively estimated MU recruitment and firing rates by means of high-density EMG and decomposition techniques. They showed that LL-BFR facilitated an early recruitment of higher threshold MUs with lower firing rates compared to LL exercise. Interestingly, the firing rate of MUs with an equal size increased after the cuff release. From a more metabolic perspective, it has been speculated that type II fiber recruitment increases with higher EMG amplitudes (Yasuda et al., 2009). Although, this cannot easily be inferred by EMG methodology, Krustrup et al. (2009) showed via muscle biopsy sampling that creatine phosphate content in type II fibers revealed a pronounced decrease compared to the same exercise without BFR. These findings underpin the statement that there seems to be a blood flow mediated feed-back which induces an increased activation of these higher threshold MU and fiber types.

#### Central Activation

The question remains, what is causing the rise of muscular activity? Generally, it has to be mentioned that the evidence describing long-term adaptations after LL-BFR on central parameters is scarce. The study by Colomer-Poveda et al. (2017) investigated the effects of 4 weeks of low-load resistance training with and without BFR on a number of neural correlates such as V-wave and H-reflex excitability. Interestingly, they did not find any significant difference between the groups in any of the variables. As H-reflexes are thought to measure spinal circuitry (even though they can be influenced by cortical pre-synaptic inhibition), it seems that changes in spinal circuits after LL-BFR training are unlikely to contribute to the observed changes in EMG. These results are confirmed by a recent meta-analysis from Siddique et al. (2020) who showed that long-term non-BFR resistance training *per se* does not seem to affect H-reflex excitability when tested at rest or during activity.

Interestingly, a study by Moore et al. (2004) demonstrated that 8 weeks of LL-BFR training resulted in changes of neural indices such as a depressed resting twitch torque or an increased post-activation potentiation (PAP). While it is not clear what was causing the decrease in resting twitch torque, the increase in PAP could be one potential mechanism explaining the increased adaptations in muscle strength after LL-BFR training compared to LL training without BFR even though no long-term study has investigated this. In an acute study, Kjeldsen et al. (2019) looked at central neural changes following a single bout of LL-BFR and LL exercise and found neither a group difference in short-interval intracortical inhibition (SICI), intracortical facilitation (ICF) nor in single pulse motor evoked potentials (MEPs) measured in the tibialis anterior muscle (TA). One reason explaining why the authors did not observe any changes in these parameters could be that they applied these measures during rest and not during activity even though it is known that especially inhibition is known to be task-specifically modulated (Soto et al., 2006; Opie and Semmler, 2016; Taube et al., 2019).

Another acute cross-over trial by Cook et al. (2013) investigated the neuromuscular function following an acute bout of LL (20% peak torque) and LL-BFR (20% peak torque) exercise to fatigue and found that both strategies did not cause significant alterations in central activation.

### LL-BFR vs. HL

#### Muscle Excitation

In the previous section we showed that long-term LL-BFR favors an increased muscle excitability compared with LL training without BFR. Whether long-term HL and LL-BFR training also differ in terms of long-term muscle excitation seems to be less clear when looking at results presented in Figure 3. There were only two studies meeting the inclusion criteria for the comparison between long-term HL and LL-BFR. After 8 weeks of HL (80% 1RM) and LL-BFR (30% 1RM) training, Ramis et al. did not find a significantly different change in VL EMG amplitude between both training regimens (Ramis et al., 2020). The study by Sousa et al. (2017) investigated the effects of 6 weeks of unilateral knee extension training. The results showed that both HL and LL-BFR significantly increased their muscle excitation in the vastus lateralis muscle without significant differences between the groups. Surprisingly, the results from their study seem to suggest that LL-BFR training causes an over 20% greater degree of muscular excitation than HL. Previous acute studies, however, indicate that the elevated EMG amplitude following LL-BFR is usually not higher than to the one observed after HL (Buckner et al., 2019; Ilett et al., 2019). In general, a tendency toward higher EMG amplitudes in HL conditions have been found (Cook et al., 2013; Buckner et al., 2019). One explanation for the higher level in knee extensor muscle excitation following LL-BFR described by Sousa et al. (2017) might be that the EMG measured after 6 weeks of training was normalized to the maximal EMG activity during the pre-test. Thus, changes in EMG amplitude were reported in relation to the pre-test. However, due to potential changes in electrode configuration, body composition, and motoneuron recruitment and firing (Patten et al., 2001; Siddique et al., 2020) typically seen after several weeks of strength training, it could be that the measures obtained after the end of the training period overestimate the actual changes in muscle excitability. A more strict and standardized normalization of the data (e.g., to maximal M-wave amplitude or maximal EMG activity of the post-test) in future studies is therefore warranted for an adequate interpretation of these variables.

#### Central Activation

In terms of central activation, two studies comparing HL with LL-BFR looked at changes in electrically evoked superimposed twitches. A study by Cook et al. (2018) compared HL resistance training (70% 1RM) with LL-BFR (20% 1RM) and failed to identify significant changes. In contrast, a study by Kubo et al. (2006) compared HL strength training at 80% 1RM with LL-BFR also at 20% 1RM and reported of a significant increase in central activation only in the HL group. Thus, it seems that adaptations in twitch torque do not seem to be pronounced following LL-BFR. So, the question remains what is causing the changes in muscle excitation seen after LL-BFR and possibly also the strength gains seen after LL-BFR? Possible explanations might come from conventional resistance training studies which were recently summarized and quantitively analyzed by Siddique et al. (2020). For example, corticospinal excitability was increased after strength training when measured during activity while silent period duration was shorter and short-interval intracortical inhibition (SICI) reduced after HL strength training (for detailed review please see Siddique et al., 2020). As the silent period and SICI are believed to be measures of inhibition, it appears that HL and maybe also LL-BFR results in reduction in inhibition which might also explain the increase in corticospinal excitability. One acute study by Brandner et al. (2015) also showed that normalized MEPs were significantly enhanced after LL-BFR compared to LL and HL training without BFR while SICI did not significantly change even though HL and LL-BFR resulted in a decrease in SICI. What is causing the reduction in inhibition after LL-BFR is not known and therefore speculation. Previous investigations have argued that group III and IV afferents might be stimulated by metabolic accumulation and subsequently inhibit the excitability of alpha motoneurons which ultimately change recruitment patterns to maintain force output (Yasuda et al., 2010). A positive correlation between metabolic accumulation and myoelectric activity following BFR exercise has also recently been shown (Centner et al., 2019a). Although this might explain the shorter times to fatigue when training with BFR (Husmann et al., 2018), it cannot account for long-term increase in MEP and the reduction in SICI. It could be that the decrease in SICI with LL-BFR is similar to the one seen after several weeks of HL (Siddique et al., 2020) resulting in a similar elevated level of excitation allowing the motor system to provide the maximal level of cortical drive (Taube et al., 2019).

## Practical Implications

The common goal of sports medicine and clinical rehabilitation is to support a quick return-to-sport of injured athletes (Kraemer et al., 2009). The topic of the present systematic review with meta-analysis is of high relevance for practitioners but also illustrate the lack of research surrounding the neural adaptations seen following low-load resistance training with blood flow restriction.

For instance, alterations in neural excitation and muscle inhibition have frequently been observed following common sports injuries including anterior cruciate ligament reconstruction (Pietrosimone et al., 2015; Harkey et al., 2016) or chronic ankle instability (McLeod et al., 2015). Furthermore, it is well acknowledged that during training or rehabilitation, neural adaptations together with structural changes within the muscle contribute to gains in force production (Sale, 1988). Exercising with blood flow restriction has been proven to be an efficient and tolerable tool in musculoskeletal rehabilitation (Hughes et al., 2017) with both functional and structural benefits compared to the same exercise under free blood flow conditions (Lixandrao et al., 2018; Centner et al., 2019b). There is, however, insufficient evidence focusing on the underlying mechanisms. From a practical standpoint, the findings from this systematic review and meta-analysis indicate that neural adaptations, especially on the EMG level, can be augmented when applying BFR to LL exercise. These results seem to support the notion that besides structural, also neural changes might contribute to the observed strength increases following LL-BFR training (Lixandrao et al., 2018; Centner et al., 2019b). An enhanced neural drive might be of special interest for practitioners or athletes who aim for an improvement in sports disciplines determined by fast and ballistic muscle contractions (e.g., rapid jumps, spring running) (Aagaard et al., 2002). Furthermore, the findings from the present work also demonstrate high relevance during the prevention of falls for older individuals to rapidly regain postural control (Aagaard et al., 2002).

## Limitations and Strengths of this Review

For an adequate interpretation of the present findings, there are some critical aspects that need to be considered. Firstly, acute and cross-sectional studies examining the effects of LL-BFR training on neuromuscular parameters are currently becoming available in large quantities (Kinugasa et al., 2006; Lauver et al., 2017, 2019; Husmann et al., 2018; Ilett et al., 2019; Kjeldsen et al., 2019). However, trials investigating long-term peripheral and central neural adaptations are scarce. For this reason and because of the strict inclusion criteria of this study, only a limited number of studies were included in this systematic review and meta-analysis. Nevertheless, the comparison between LL-BFR and LL on EMG amplitude allowed a calculation of a meta-analysis, which aimed to combine the results of multiple individual reports and create a more precise effect estimate by improving statistical power (Hoffman, 2015). This, however, was not possible for the comparison between LL-BFR and HL training due to insufficient data availability. Since only two studies were found to be eligible to do a quantitative analysis and because these studies also demonstrated a high heterogeneity (*I*^2^ = 67%), we decided to omit the pooled effect size in order to prevent misinterpretation of the forest plot (Figure 3). With the descriptive presentation of the forest plot in Figure 3, we aim to make aware of the current conflicting findings between studies. For both meta-analyses, only studies with parallel-group designs were included because within-subject designs result in smaller variances and might also be biased by cross-education effects in training interventions (Lee and Carroll, 2007).

Secondly, most studies used surface EMG to infer that neural adaptations have occurred. Even though surface EMG is limited in its explanatory power and a rather superficial method to measure global activation of most likely superficial motor units (Vigotsky et al., 2018), it does provide an rating about the general muscle excitation changes with BFR.

Finally, systematic reviews are often flawed by integrating studies with poor methodological quality. This inevitably results in a limited validity, as described by the garbage-in-garbage-out phenomenon (Borenstein et al., 2009). To counteract this issue, only randomized-controlled trials were included and subjected to a quality assessment. During this assessment, studies with a score lower than 6 out of 10 on the PEDro scale were excluded from the systematic review. This cut-off point is substantially higher than that of previous meta-analyses in this field (Lixandrao et al., 2018; Centner et al., 2019b). A major problem in studies investigating the effects of BFR training remains the lack of participant blinding. Condition blinding is often not feasible in this research field.

## Conclusion

In summary, the present systematic review and meta-analysis provides novel insights into the neural adaptations following LL-BFR. Our quantitative approach revealed that long-term LL-BFR results in a higher muscle activation compared to LL training without BFR. In fields of central activation and corticospinal excitation, the evidence is less clear due to the small amount of studies available. The comparison to HL training indicated a tendency toward higher increases in voluntary activation following HL with two studies reporting 3% increase for HL and a non-significant ~2% decrease for LL-BFR training (Kubo et al., 2006; Cook et al., 2018). Both studies provide first evidence about the potential central adaptations following LL-BFR and the results are corroborated by EMG data between HL and LL-BFR. Further adequately powered studies, however, are urgently needed

In general, there is also a vital need for more long-term studies using sophisticated measurements of neural parameters (e.g., intracortical inhibition, cortical facilitation, single motor unit activity) to further investigate the occurring adaptations following LL-BFR training. Since this systematic review incorporated longitudinal studies with a duration of 1–3 months, future BFR research is warranted to further investigate even longer lasting adaptations (several months or years). Since it was beyond the scope of this review to examine BFR specific parameters (intermittent vs. continuous, cuff pressure, etc.), future studies are needed to examine the influence of such moderator variables. Additionally, the results of this review rely on studies investigating neural modifications in healthy individuals and must therefore not be transferred to clinical populations.

## Data Availability Statement

All datasets generated for this study are included in the article/Supplementary Material.

## Author Contributions

CC and BL contributed to preparation, literature identification, and analyses presented in the current systematic review.

## Conflict of Interest

The authors declare that the research was conducted in the absence of any commercial or financial relationships that could be construed as a potential conflict of interest.

## Supplementary Material

Supplementary Material 1 Risk of bias for each study.

Supplementary Material 2 Funnel plot of the comparison between the effects of low-load BFR and low-load training without BFR on muscle excitation. SE (SMD), standard error of SMD; SMD, standardized mean difference.

Supplementary Material 3 Funnel plot of the comparison between the effects of low-load BFR and high-load training on muscle excitation. SE (SMD), standard error of SMD; SMD, standardized mean difference.
