# Supplementary material for: A Systematic Review and Meta-Analysis on Neural Adaptations Following Blood Flow Restriction Training: What We Know and What We Don't Know
Source: Front Physiol. 2020 Aug 4;11:887. doi: 10.3389/fphys.2020.00887 (PMC7417362; doi:10.3389/fphys.2020.00887)
Supplement: Supplementary Material 1 — Risk of bias for each study. [file Data_Sheet_1.pdf]

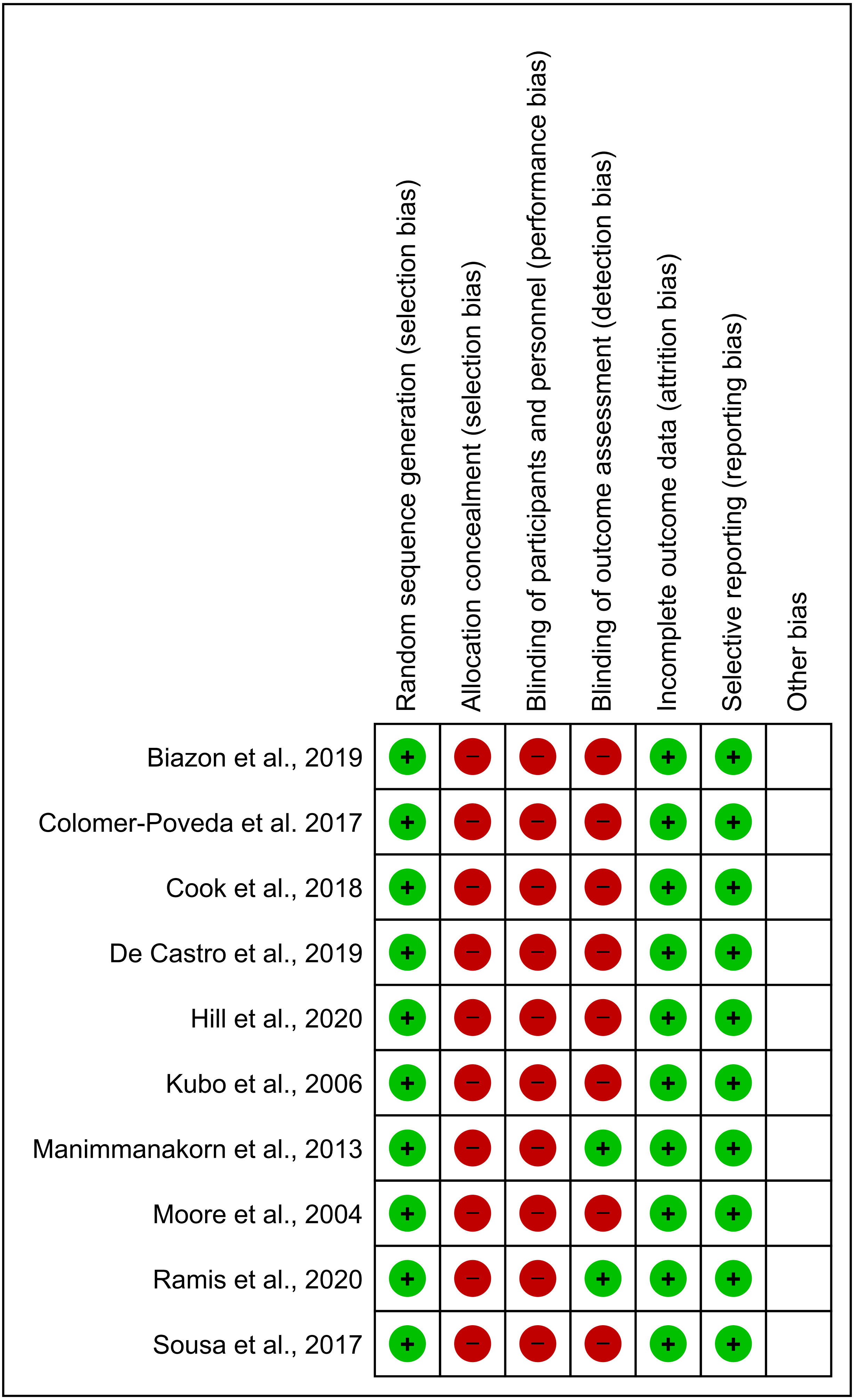
Random sequence generation (selection bias)
Allocation concealment (selection bias)
Blinding of participants and personnel (performance bias)
Blinding of outcome assessment (detection bias)
Incomplete outcome data (attrition bias)
Selective reporting (reporting bias)
Other bias
Biazon et al., 2019
Colomer-Poveda et al. 2017
Cook et al., 2018
De Castro et al., 2019
Hill et al., 2020
Kubo et al., 2006
Manimmanakorn et al., 2013
Moore et al., 2004
Ramis et al., 2020
Sousa et al., 2017
