# Supplementary figures and images for: A Systematic Review and Meta-Analysis on Neural Adaptations Following Blood Flow Restriction Training: What We Know and What We Don't Know
Source: Front Physiol. 2020 Aug 4;11:887. doi: 10.3389/fphys.2020.00887 (PMC7417362; doi:10.3389/fphys.2020.00887)

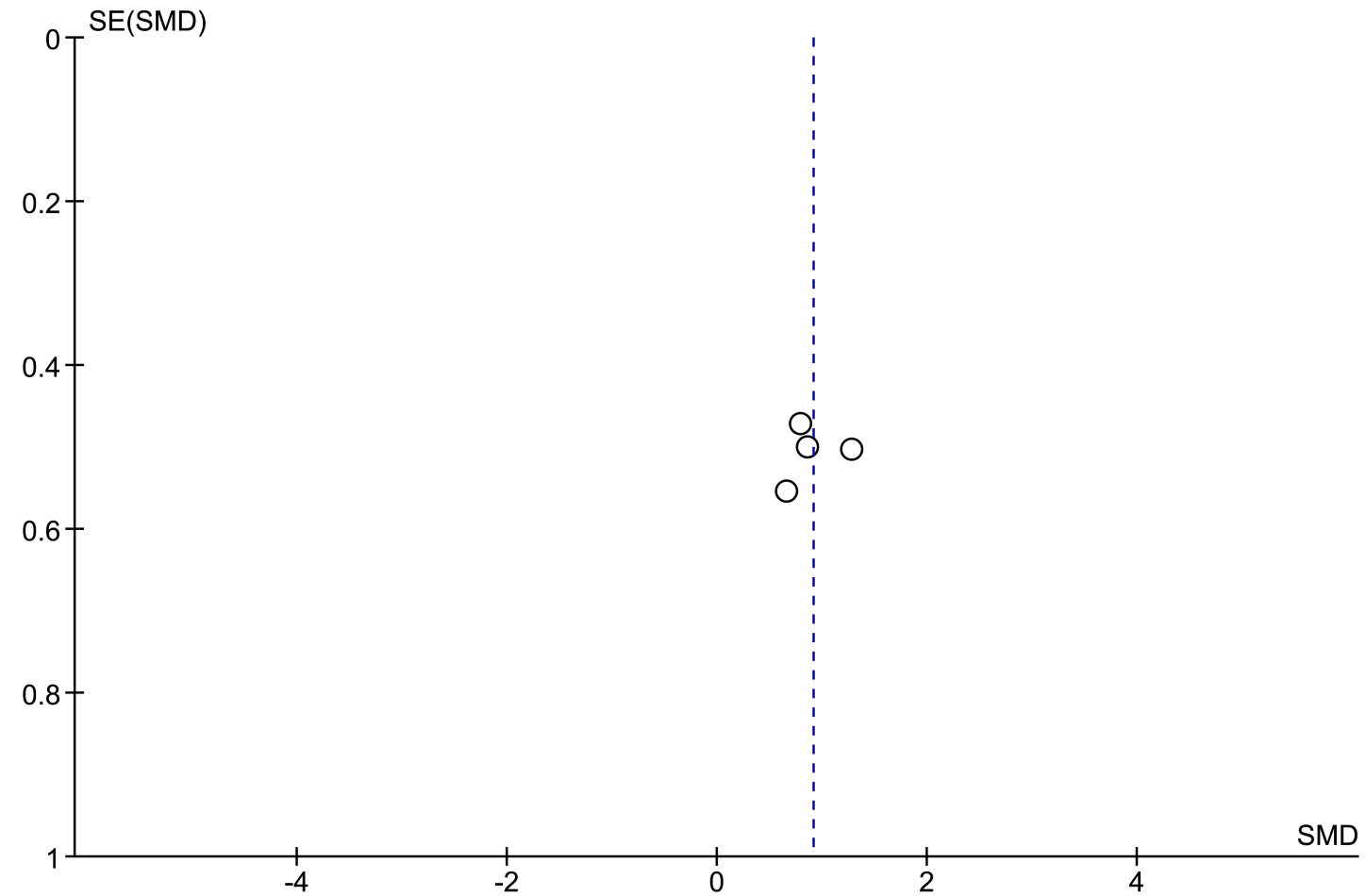

Supplement: Supplementary Material 2 — Funnel plot of the comparison between the effects of low-load BFR and low-load training without BFR on muscle excitation. SE (SMD), standard error of SMD; SMD, standardized mean difference. [file Data_Sheet_2.PDF]

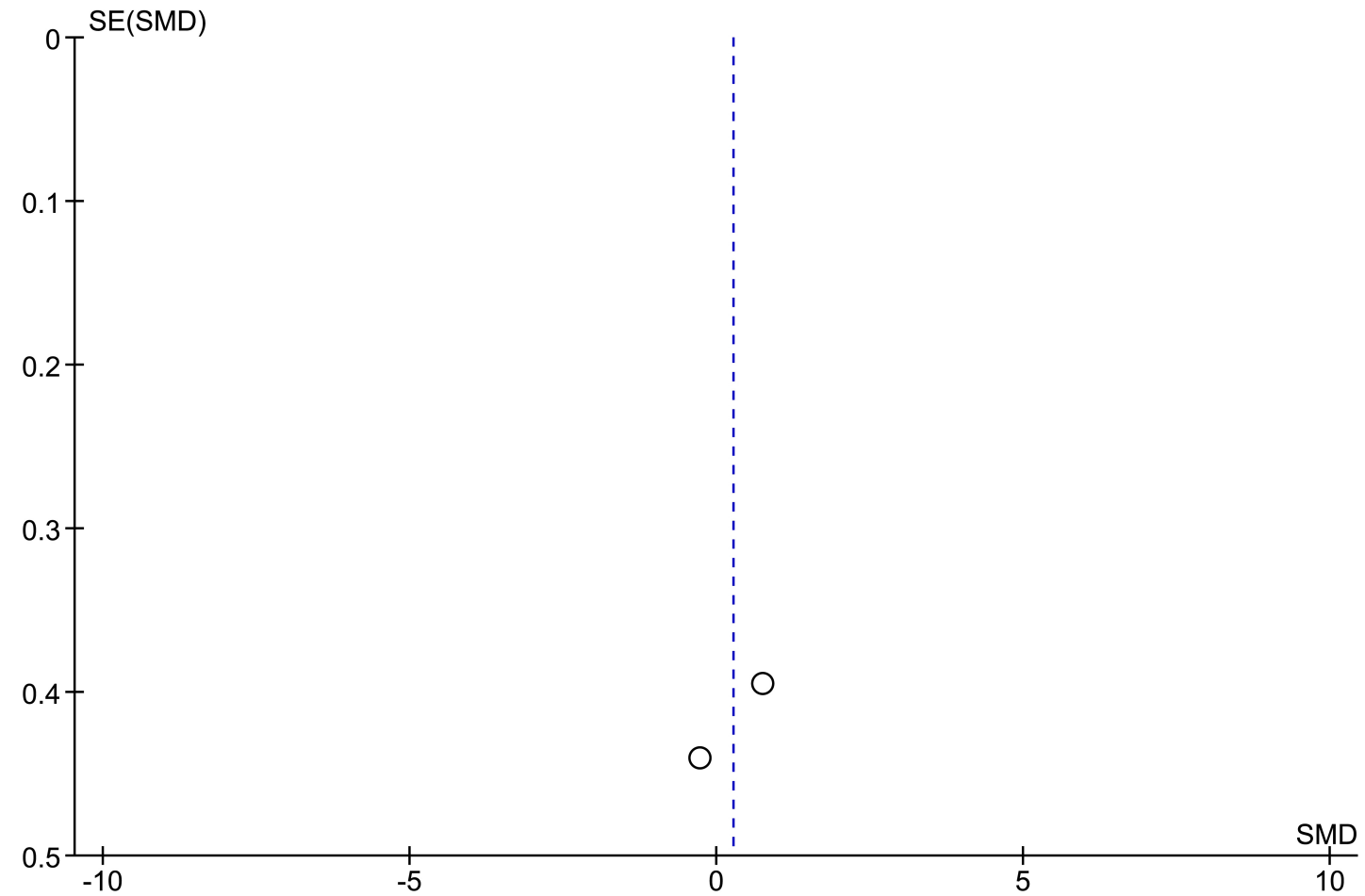

Supplement: Supplementary Material 3 — Funnel plot of the comparison between the effects of low-load BFR and high-load training on muscle excitation. SE (SMD), standard error of SMD; SMD, standardized mean difference. [file Data_Sheet_3.PDF]
